# Assessing the Safety, Tolerability and Efficacy of Cell‐Free Amniotic Fluid in the Treatment of Non‐Healing Venous Ulcers: Initial Experience From a Prospective, Multicenter, Phase II Study

**DOI:** 10.1111/iwj.70171

**Published:** 2025-03-24

**Authors:** Frederick Ditmars, Sarah E. Ducharme, Aliza M. Lee, Jo‐Anna Reems, William Samuel Fagg, Jovan N. Markovic

**Affiliations:** ^1^ Department of Internal Medicine University of Pennsylvania Philadelphia Pennsylvania USA; ^2^ Department of Dermatology University of Pennsylvania Philadelphia Pennsylvania USA; ^3^ Compass Medical Research Center Tucson Arizona USA; ^4^ Department of Podiatry Salem Veterans Affairs Health Care System Salem Virginia USA; ^5^ Merakris Therapeutics Inc., Research Triangle Park Durham North Carolina USA; ^6^ Department of Surgery University of Texas Medical Branch Galveston Texas USA; ^7^ Department of Surgery Duke University Medical Center Durham North Carolina USA

**Keywords:** amniotic fluid, chronic wound, regenerative medicine, venous leg ulcer, wound healing

## Abstract

Non‐healing venous leg ulcers represent a significant healthcare problem that accounts for about $32 billion of spending in the US alone. Consequently, novel treatment strategies represent a major unmet need. The current study (part one of Phase II study [NCT04647240]) assesses the safety, tolerability and efficacy of the use of cell‐free human amniotic fluid in treating venous leg ulcers that did not heal following the correction of venous reflux. Patients received cell‐free amniotic fluid injections in and around the wound either weekly or biweekly over 12 weeks. Primary outcomes included safety, tolerability and efficacy assessed by complete wound closure, wound area reduction and pain reduction. Eleven patients met enrollment eligibility, and nine completed the study. Five patients achieved complete wound closure by week 12. The average percent reduction in wound area was 83.7%, and pain scores were significantly lower by the study endpoint. No difference was observed in wound healing rates between weekly or biweekly treatment, but bi‐weekly treatment was associated with nominally faster recovery. Patients tolerated the treatment, and no side effects were reported. These results indicate that cell‐free amniotic fluid injection is a feasible, safe and effective treatment for non‐healing venous leg ulcers.

**Summary**

- New therapeutic modalities are required to efficiently and successfully treat non‐healing and/or recurring venous leg ulcers. Amniotic membranes have been used in this setting, but it is not clear if cell‐free amniotic fluid can also promote successful healing, outside of an anecdotal report. In the first part of this Phase II clinical trial, we find that the use of cell‐free human amniotic fluid is safe, effective and well‐tolerated, over a 12‐week period in a cohort of nine patients. We conclude that this simple but novel regenerative treatment can successfully treat non‐healing venous leg ulcers, which should significantly reduce the overall healthcare burden if it can be widely adopted.

## 1 Introduction

Chronic venous insufficiency (CVI) impacts a substantial portion of the population [1, 2]. Approximately 25% of women and 15% of men experience symptoms and signs of CVI, with up to 3% of American adults suffering from the most severe stages of the disease, including chronic, non‐healing venous leg ulcers (VLU) (Class C6; Clinical Severity, Aetiology or Cause, Anatomy, Pathophysiology [CEAP] classification) [3, 4]. The estimated incidence of VLU is even higher in individuals older than 65, with reported rates of up to 5% [5]. Although approximately 60% of VLUs heal within 12 weeks, the rates of recurrence are alarmingly high and range from 30% to 70% after 1 or 2 years, respectively [6].

The impact on health‐related quality‐of‐life (HR‐QoL) for patients with non‐healing VLUs is severe. The chronic nature of the disease and its associated symptoms and complications, such as pain, haemorrhage, infections, malodor, mobility restrictions, and a reduced ability to perform daily activities, contribute to a continuous cycle of physical and emotional suffering [7, 8, 9]. Consequently, high rates of depression and social isolation are reported for this patient population [10].

Sequential ulceration and recurrence not only reduce the patient's HR‐QoL but also place a considerable strain on healthcare resources. Given patients' needs for repeated hospitalizations and advanced wound care, VLU treatments cost ~$3.5 billion annually, with Medicare expenditure alone accounting for over $1.13 billion in 2019 (~1.9‐fold increase over a 5‐year period) [11, 12, 13]. The major driver of these expenses is chronically ulcerated VLUs: they disproportionally impact these expenses, with threefold higher mean treatment costs compared with healing/non‐chronic VLUs ($33 907 vs. 10 563) [14]. Indirect costs, such as loss of productivity due to prolonged treatment and recovery periods, further compound the economic burden [15]. Finally, it is important to note that the prevalence of CVI is rising due to increased life expectancy and the aging of the “baby boomer” generation, amplifying its impact on healthcare systems. Thus there is a major need for innovative treatment strategies that mitigate these negative impacts of chronic, non‐healing VLUs.

Cell‐free human amniotic fluid (cfAF) is a regenerative biologic that has great promise in the treatment of wounds and other fibrotic diseases [16]. Full‐term cfAF contains a variety of biomolecules that can be either freely soluble or encapsulated in extracellular vesicles, which can alter cell signalling and gene expression in target cells [17, 18]. While various studies indicate that amniotic membrane (AM) can promote VLU healing [19, 20, 21, 22, 23, 24], we have observed successful treatment of VLUs that had persisted for over 2 years in a single patient after combined administration of AM and cfAF [25]. However, it remains an open question if cfAF as a standalone treatment is safe and effective in a VLU patient cohort greater than *n* = 1. Thus, we undertook the first part of a Phase II clinical trial to assess the safety and efficacy of cfAF in the treatment of uninfected non‐healing VLUs that persist despite correction of the underlying venous pathology.

## 2 Methods

### 2.1 Study Design

This report is the first part of a prospective, multicenter, two‐part, Phase II clinical trial (NCT04647240) of patients with non‐healing, non‐infected, refractory to SOC VLU who underwent treatment with cfAF (Dermacyte Liquid, Merakris Inc., Durham, NC, USA). Patients were randomised 1:1 to receive this once weekly or every other week over a 12‐week course. During their course, subjects were assessed at baseline (Week 0) and evaluated at weeks 4, 8 and 12 (±7 days). These evaluations included screenings for adverse events, wound size changes, pain scores, and infection. This study was conducted under IRB #20213102 and approved by the Western Copernicus Group Institutional Review Board. A written signed informed consent was obtained for each subject.

### 2.2 Inclusion Criteria

Eligible female and male subjects needed to be between the ages of 18 and 75 years with full‐thickness VLU. Ulcers with surface areas ranging in size from 1 to 25 cm^2^ and > 0.2 cm deep at the deepest point of the wound were included in the study. All the subjects were required to have previously undergone treatment to correct their underlying venous reflux disease to be eligible for the study; this includes sclerotherapy, graded compression, surgical venous stripping, endovenous laser ablation and/or radiofrequency ablation. Underlying venous reflux disease was confirmed by a Doppler ultrasound using the following criteria: superficial veins reflux > 500 ms (0.5 s), deep veins reflux > 1000 ms (1 s), and perforator reflux > 500 ms (0.5 s). Selected subjects were required to have received > 28 days of standard conventional wound therapy with graduated‐compression, multilayer bandaging before a baseline visit. Subjects were required to have an ankle‐brachial index (ABI) of ≥ 0.8 and ≤ 1.2 or triphasic or biphasic Doppler arterial waveforms at the ankle of the affected leg. Female subjects of childbearing age were required to have a negative urine pregnancy test. Subjects were also required to answer surveys and questionnaires written in English.

### 2.3 Exclusion Criteria

Subjects were excluded from the study if their VLU was infected or if they were receiving topical antimicrobial treatment for ulcer infection at the time of enrollment. VLU could not have exposed bone, tendon, or ligament or have another ulcer within 3 cm. Subjects at the time of screening could not be actively receiving the following treatments within 30, 60, and 90 days of cfAF treatment: (1) a skin graft substitute within 30 days of start date; (2) corticosteroid therapy within 60 days of study initiation and/or (3) an investigation drug or device within 90 days prior. Subjects with the following medical history were excluded from the study (1) peripheral arterial disease (PAD); (2) congestive heart failure; (3) osteomyelitis; (4) active infection; (5) uncontrolled diabetes mellitus (HbA1c > 7.0% within the last 120 days); (6) chronic musculoskeletal disorders; (7) a history of alcohol or drug abuse within 12 months at the time of screening; (8) a diagnosis of a concomitant disease with a life expectancy of < 12 months; (9) an unstable psychiatric condition.

### 2.4 Administration of cfAF

**FIGURE 1:**
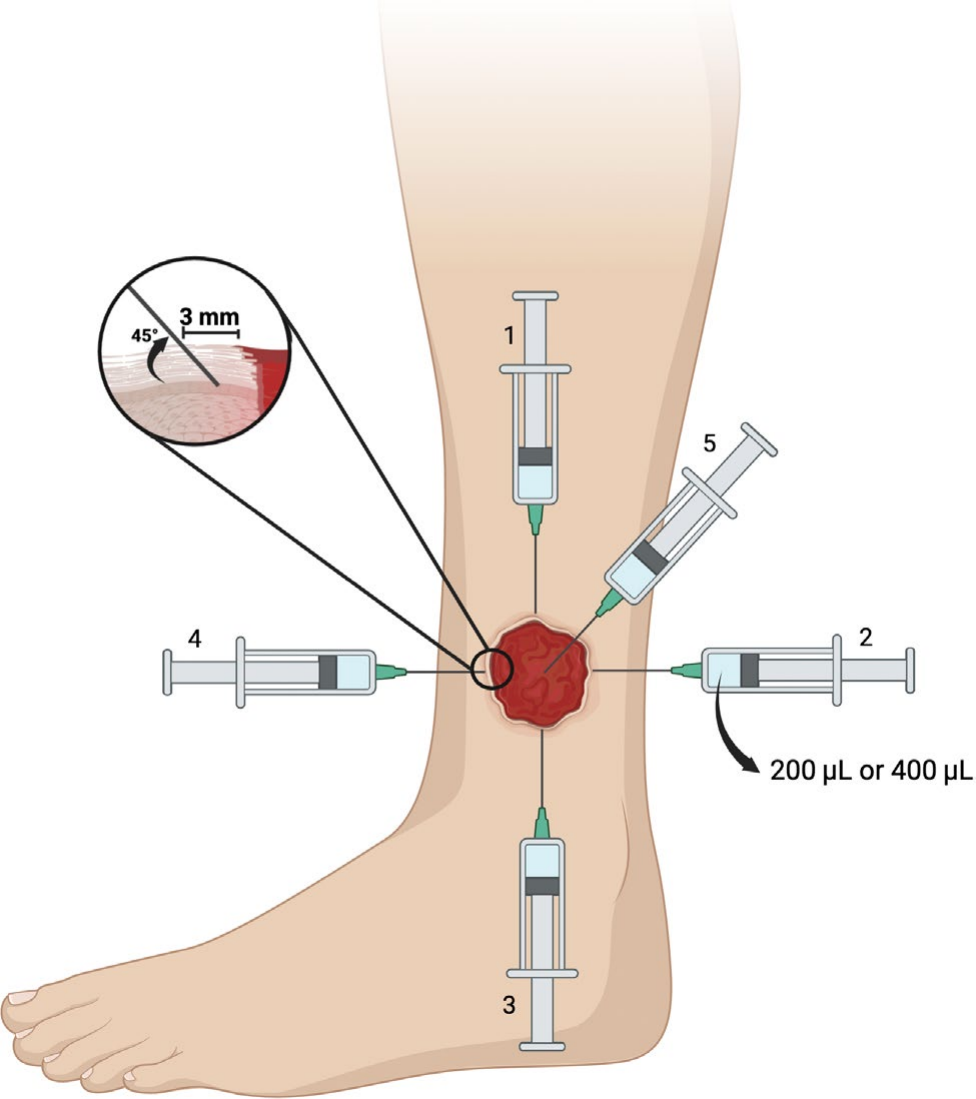
Overview of the cfAF injection strategy. A graphical representation illustrating the administration of cfAF, as described in the Methods section. Created in BioRender. Ditmars et al. [21] https://BioRender.com/o03d205.

VLUs ≤ 12.5 cm^2^ were treated with 1 mL, and ulcers > 12.5 and ≤ 25 cm^2^ received 2 mL of cfAF. Subcutaneous cfAF injections with a needle positioned at a 45° angle were administered as follows: circumferentially into healthy appearing dermal tissue at 12‐, 3‐, 6‐ and 9‐o'clock positions 3 mm lateral of the VLU margin followed by a single injection into the middle of the VLU (Figure 1). Patients receiving 1 mL of cfAF were treated with five injections of 200 μL each per treatment visit, and patients receiving 2 mL were treated with five injections of 400 μL each per treatment visit.

### 2.5 Objective(s)

The primary goal of the study was to assess tolerability and safety and to gain preliminary insights into the efficacy of cfAF. The secondary goal was to identify the frequency at which to administer cfAF. Efficacy endpoints were complete wound closure, VLU area percent reduction, pain reduction, and health‐related quality of life (HR‐QoL). To assess wound healing, each VLU was evaluated by measuring the wound surface area at the baseline and then at each follow‐up visit. A percent reduction in wound size was calculated using the formula (Area_t0_ − Area_ti_)/Area_t0_ × 100 (where ti = baseline measurement and ti = measurements at 4, 8, or 12 weeks). Patient‐reported VLU‐related pain was measured by using the Visual Analogue Scale (VAS; 0 = no pain, 5 = moderate pain, and 10 = worst pain). Changes in HR‐QoL from baseline to week 12 were assessed by using the short form‐36 health survey (SF‐36). The dermatology life quality index (DLQI) and the wound quality of life (wQoL) self‐administered questionnaires were also used to assess changes in the subject's QoL. The safety was determined by monitoring for any adverse events by a treating physician, and any adverse events or their absence were documented. The secondary endpoint was determined by comparing outcomes between bi‐weekly and weekly cfAF administration regimens.

### 2.6 Statistics

Descriptive statistics were used for complete would closure and VLU area percent reduction analysis. Statistical analysis and graphs were made using the Graphpad Prism 10 software (Boston, MA). Wound size statistical tests were done using a mixed effect model with Dunnett's multiple comparison test, with wound size at week 0 serving as our comparison group. Mixed effect analysis was done via multiple linear regressions, calculating a Pearson's *R*‐value for each of the five factors measured. Descriptive statistics and measures of variation were calculated using Excel. Paired samples *t*‐tests were used for Descriptive pain reduction and HR‐QoL outcomes (SF‐36, DLQI, and wQoL). Statistical significance was set at *p* < 0.05.

## 3 Results

Eight males (73%) and three females (27%) ranging in age from 41 to 73 years (63.75 ± 9.99 years [mean ± SD]) met enrollment eligibility. Nine patients (six males and three females) with an average age of 66.76 ± 6.96 [mean ± SD] completed the 12‐week series of cfAF injections. Two patients were withdrawn: one due to infection and the other for non‐compliance. All of the wounds were classified as C6 based on the internationally recognised classification system of CEAP (Clinical‐Aetiology‐Anatomy‐Pathophysiology) (open, non‐healing ulcers) [4]. All of the patients had previously undergone endovenous ablation for their ultrasound‐confirmed underlying venous reflux disease. Following ablation, each patient received SOC wound treatment with high compression and multilayer bandaging for at least 28 days before initiating cfAF treatment.

The majority of the enrolled patients were White (72% enrolled; [56% completed the 12‐week trial]), with the remaining patients being Native American, Asian or Black (9%; [11%]). Of the White patients, 33% [28.5%] was identified as Hispanic or Latino. Moreover, the majority of the enrolled patients were male (73%; [67%]), despite the predominance of females with VLUs described in the literature (Table 1) [26]. The median participant's BMI was 34, with 73%; [66%] meeting the criteria for obesity. Patients had a myriad of other complicating comorbidities at the time of enrollment, including diabetes (45%; [44%]), hypertension (64%; [78%]), hyperlipidemia (45%; [56%]), osteoarthritis (27%; [22%]) previous DVT (36%; [44%]) or other, less common comorbidities (Table 2).

**TABLE 1:** Demographics.

Characteristic	*N*	Overall, *N* = 11^a^	Completed 12‐week trial		*p* ^b^
			N, *N* = 2^a^	Y, *N* = 9^a^	
Age	11	65 (58, 72)	50 (46, 55)	69 (63, 73)	0.15
*Gender*					
F	11	3 (27%)	0 (0%)	3 (33%)	> 0.99
M		8 (73%)	2 (100%)	6 (67%)	
*Race*					
Asian/Pacific Islander	11	1 (9.1%)	0 (0%)	1 (11%)	> 0.99
Black		1 (9.1%)	0 (0%)	1 (11%)	
Native American		1 (9.1%)	0 (0%)	1 (11%)	
White		8 (73%)	2 (100%)	6 (67%)	
*Ethnicity*					
Hispanic/Latino	11	3 (27%)	1 (50%)	2 (22%)	0.49
Not Hispanic/Latino		8 (73%)	1 (50%)	7 (78%)	

^a^ Median (IQR); *n* (%).

^b^ Wilcoxon rank‐sum exact test; Fisher's exact test.

**TABLE 2:** Comorbidities.

Characteristic	*N*	Overall, *N* = 11^a^ (%)	Completed 12‐week trial (%)		*p* ^b^
Obesity	11	6 (55)	1 (50)	5 (56)	> 0.99
Diabetes	11	5 (45)	1 (50)	4 (44)	> 0.99
Hypertension	11	7 (64)	0 (0)	7 (78)	0.11
HLD	11	5 (45)	0 (0)	5 (56)	0.45
Glaucoma	11	2 (18)	0 (0)	2 (22)	> 0.99
OA	11	3 (27)	1 (50)	2 (22)	0.49
A.Fib	11	1 (9.1)	0 (0)	1 (11)	> 0.99
PAD	11	1 (9.1)	0 (0)	1 (11)	> 0.99
DVT	11	4 (36)	0 (0)	4 (44)	0.49
F5 Leiden	11	1 (9.1)	0 (0)	1 (11)	> 0.99
Smoker	11	1 (9.1)	0 (0)	1 (11)	> 0.99
Lymphedema	11	1 (9.1)	0 (0)	1 (11)	> 0.99
HyperT4	11	1 (9.1)	0 (0)	1 (11)	> 0.99
HypoT4	11	2 (18)	0 (0)	2 (22)	> 0.99
OSA	11	1 (9.1)	0 (0)	1 (11)	> 0.99
Previous Malignancy	11	2 (18)	1 (50)	1 (11)	0.35
RA	11	1 (9.1)	0 (0)	1 (11)	> 0.99
Lupus	11	1 (9.1)	0 (0)	1 (11)	> 0.99
Osteoporosis	11	1 (9.1)	0 (0)	1 (11)	> 0.99
CHF	11	2 (18)	0 (0)	2 (22)	> 0.99
Polycythemia	11	1 (9.1)	0 (0)	1 (11)	> 0.99
CKD	11	1 (9.1)	0 (0)	1 (11)	> 0.99
Erectile dysfunction	11	1 (9.1)	1 (50)	0 (0)	0.18
Insomnia	11	1 (9.1)	1 (50)	0 (0)	0.18
GERD	11	1 (9.1)	1 (50)	0 (0)	0.18
COPD	11	1 (9.1)	0 (0)	1 (11)	> 0.99
Asthma	11	1 (9.1)	0 (0)	1 (11)	> 0.99

^a^ Median (IQR); *n* (%).

^b^ Wilcoxon rank‐sum exact test; Fisher's exact test.

The median wound duration was 213 days (interquartile range [IQR]: 192, 647) for those enrolled and 208 (IQR: 179, 399) in those who received 12 weeks of treatment. Initial wound size was a median 7.2 cm^2^ (IQR: 4.9, 16.8) in the enrolled and 7.2 cm^2^ (IQR: 5.0, 13.6) in those who completed the trial. Statistically, there was no difference in wound size or duration when comparing all who enrolled to those who completed the 12‐week trial (Table 3).

**TABLE 3:** Wound and treatment characteristics.

Characteristic	*N*	Overall, *N* = 11^a^	Completed 12‐week trial		*p* ^b^
			N, *N* = 2^a^	Y, *N* = 9^a^	
Wound duration (days)	11	213 (192, 647)	1287 (1073, 1501)	208 (179, 399)	0.073
Wound size (CM2)	11	7.2 (4.9, 16.8)	14.9 (9.9, 19.9)	7.2 (5.0, 13.6)	0.72
*Wound initial size*					
< 12.5	11	7 (64)	1 (50)	6 (67)	> 0.99
> 12.5		4 (36)	1 (50)	3 (33)	
*Wound duration*					
< 24 Weeks	11	1 (9.1)	0 (0)	1 (11)	> 0.99
> 24 Weeks		10 (91)	2 (100)	8 (89)	
*Site*					
Left ankle	11	2 (18)	0 (0)	2 (22)	> 0.99
Left leg		2 (18)	1 (50)	1 (11)	
Right ankle		2 (18)	0 (0)	2 (22)	
Right leg		5 (45)	1 (50)	4 (44)	
*Weekly/biweekly*					
Biweekly	11	7 (64)	1 (50)	6 (67)	> 0.99
Weekly		4 (36)	1 (50)	3 (33)	

^a^ Median (IQR); *n* (%).

^b^ Wilcoxon rank‐sum exact test; Fisher's exact test.

**FIGURE 2:**
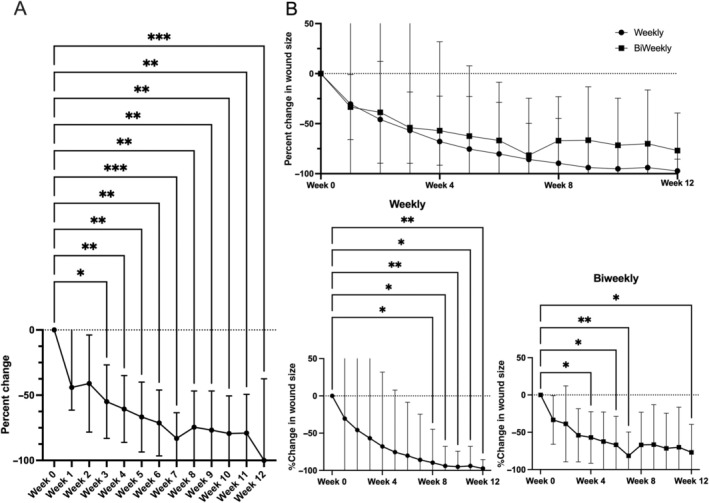
Changes in wound size in patients who completed the 12‐week series of cfAF injections. (A) Mean wound size reduction as a percentage change in total wound size over time. (B) Mean wound size reduction as above, but separated by dosage frequency; bottom insets show individual mean values for weekly (left) or biweekly (right) treatment frequency (*p < 0.05, **p < 0.01, ***p < 0.001 by mixed effect model with Dunnett's multiple comparison test).

Five of the nine patients who completed 12 weeks of treatment achieved complete wound closure and re‐epithelization (56% closure rate). An additional patient achieved wound closure and re‐epithelization at week 13, which was reported within the one‐month follow‐up period. The average time to re‐epithelization among the five patients whose wounds resolved within the trial period was 8.4 ± 1.34 weeks. All patients who completed the trial saw significant reductions in wound size, with an average of 83.7% ± 10.0 reduction in the wound surface area by the study's end (*p* < 0.001 by mixed effect model with Dunnett's multiple comparison test). This rapid rate of reduction was, on average 0.896 cm^2^/week or an 11.14% weekly reduction in the initial wound size. Statistically significant reductions in wound size were achieved after only 3 weeks of treatment (Figure 2A).

**FIGURE 3:**
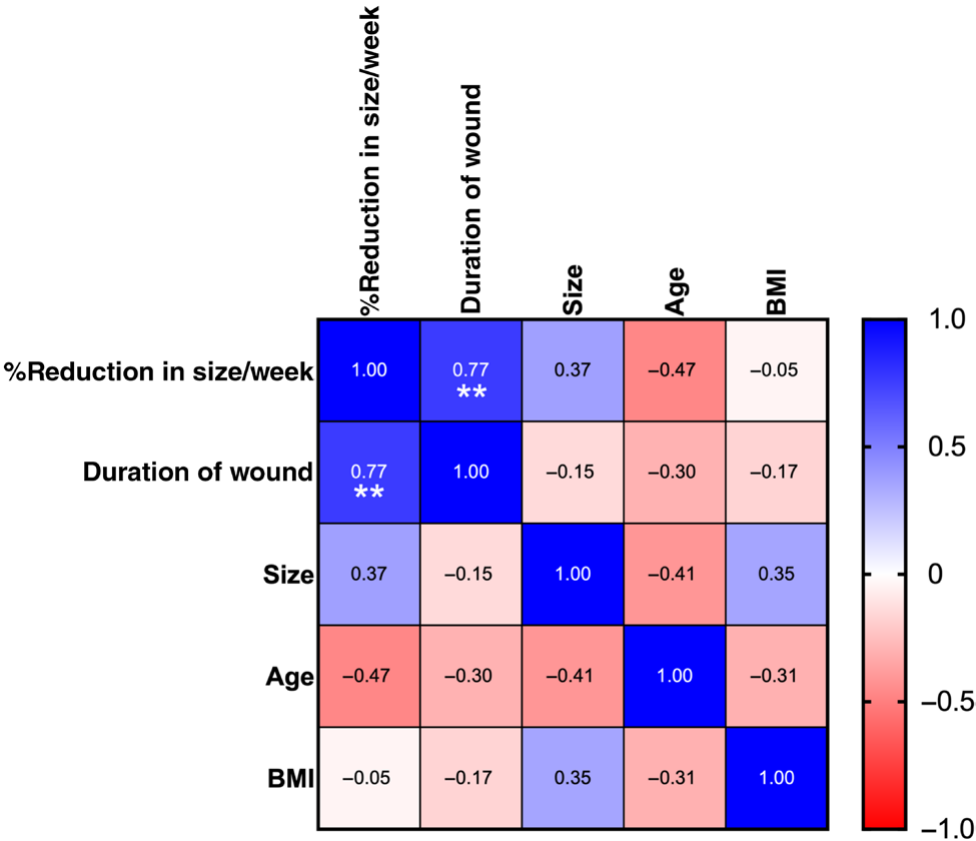
Multifactor correlation of characteristics of patients who completed a 12‐week trial of cfAF injections. Values represent Pearson's correlation *R*‐values; **p < 0.01 by multiple linear regressions using Pearson's *R*.

**FIGURE 4:**
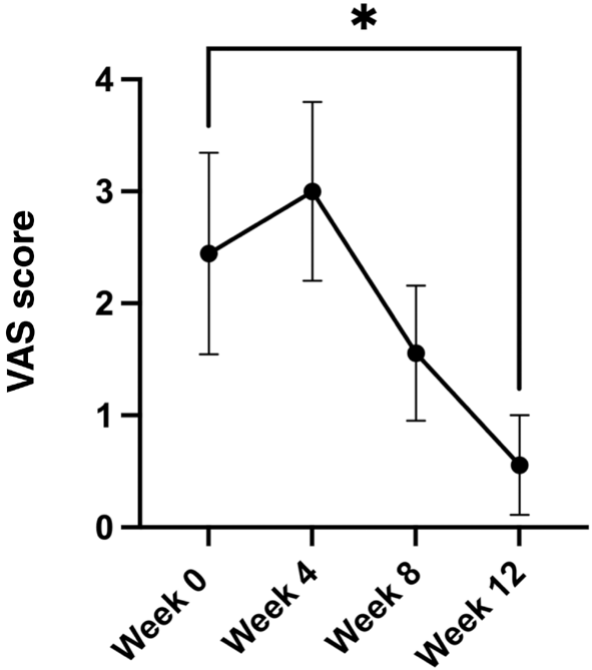
Visual Acuity Scale pain scores of patients who completed the 12‐week treatment course. Mean values are shown with error bars representing the standard error of the mean. *p < 0.05 by mixed effects model with Dunnett's multiple comparison test.

There were no significant differences in wound healing rates between the groups receiving weekly or biweekly administration of cfAF. However, those receiving weekly dosage did have a nominally faster recovery (weekly: 14.9% reduction vs. biweekly: 9.2% reduction in wound volume) (Figure 2B–D). More patients treated in this same manner will be required to determine if this difference is significant. The majority of the demographic and wound factors we measured had little to no effect on the rates of wound healing, with the exception of wound healing rate compared with wound duration. Specifically, wounds that had persisted for longer before the initiation of this study healed faster than wounds with a shorter duration (Figure 3; *p* < 0.05, Pearson *r* = 0.77, multiple linear regression). Finally, there was a statistically significant reduction in pain measured by VAS score at 12 weeks when compared with baseline (Figure 4, VAS score 2.4 ± 2.7 at week 0 vs. 0.56 ± 1.33 at week 12, *p* < 0.05 by mixed effect model with Dunnett's multiple comparison test).

Adverse events were uncommon, with no serious life‐threatening events reported or observed. One patient developed cellulitis, which resolved following oral antibiotics and did not interrupt the treatment frequency or trial participation. Two patients developed wound site infections during the trial and could not complete the treatment per trial protocol. One of these was resolved with oral and topical antibiotics, while the other was lost to follow‐up immediately after the diagnosis.

## 4 Discussion

Data from this first part of a two‐part Phase II clinical trial demonstrate both the efficacy and safety of cfAF injections in patients with chronic, difficult‐to‐treat lower extremity venous wounds. Of those who completed the 12‐week treatment course, 55% had full resolution of VLU with re‐epithelization during the treatment period with 66.7% resolving during our 1‐month post‐trial follow‐up period. Of those who did not re‐epithelize, 89% showed at least a 25% reduction in wound size. Therefore, since the wounds treated in this study had an average time of persistence of 73 weeks before the start of the study, these outcomes are very encouraging. Notably, 80% of VLU patients report some degree of pain, with an average VAS of 4/10 [27]. The patients enrolled in this study reported similar pain levels at baseline, with a statistically significant reduction in those who completed all of the visits (Figure 3). Adverse effects over the course of this trial were relatively minor and included infection and cellulitis, which resolved with a standard treatment course of oral antibiotics. Since a single lot of cfAF was used for all 11 of the patients treated in this study, and only two developed a wound infection, it is extremely unlikely that the cfAF was the source of the infection. Moreover, while wound site infection is unfortunate, it is a common complication of open ulceration: reported wound infection rates vary between 30% and 84% at any given time [28, 29, 30]. Of note is that the use of cfAF was safe and well‐tolerated in this patient cohort, as was observed in our previous case study [25].

While the results presented here are encouraging, this study has limitations. One of the primary ones is the absence of a control group, which restricts our ability to attribute observed outcomes solely to the intervention. However, our inclusion criteria aimed to include treatment‐resistant, non‐healing wounds that were unlikely to resolve. Not surprisingly, at this point in the study, we have no long‐term data; the second part of this study will include a control group and long‐term follow‐up studies. In addition, the limited sample size prevents the generalizability of our findings to a broader population and yields lower statistical power. Thus, we have observed some positive trends here that fail to meet the threshold for statistical significance. For example, we observed few predictive demographic factors that influenced the speed of wound healing, which could be due to a lack of power. In addition, none of the patients in this study had a body mass index (BMI) within the normal range: 73% were classified as obese (BMI > 30.0) and 46% as morbidly obese (BMI > 40). Previous reports indicate that recalcitrant VLUs exhibit prolonged healing times in obese individuals [8] thus, an identical study with greater power could address how cfAF treatment of VLU could be influenced by obesity. Interestingly, we did observe a direct relationship between wound duration (before the start of the study) and rates of healing, suggesting that older wounds healed faster than more recent wounds. This finding seems paradoxical but could be explained by cfAF‐mediated repression of TGFβ‐activated epithelial‐mesenchymal transition (EMT) and myofibroblast activation (MFA) [18]. Chronic wounds, in particular those following endovascular correction of reflux, may be driven by chronic inflammation and regranulation [31]. Therefore, repression of MFA and the EMT but promotion of the mesenchymal‐epithelial transition by cfAF treatment of the VLU could explain in part the mechanistic underpinnings of this positive result, in addition to why most wounds re‐epithelized instead of forming a scar. Further investigations will address these limitations and dissect the molecular, cellular and biochemical mechanisms through which cfAF promotes chronic wound healing.

To our knowledge, this is the first trial outside of a *n* = 1 case study to evaluate the safety, tolerability and efficacy of cfAF treatment of chronic, non‐healing VLUs. The data from part one of this Phase II study indicate that cfAF treatment in VLU patients with corrected underlying venous reflux disease is well‐tolerated and effective, with acceptable complication rates. Overall, this study suggests that irrespective of weekly or biweekly administration, cfAF can effectively treat advanced VLUs, ultimately reduce clinic visits, and improve patient QoL.

## Conflicts of Interest

J.‐A.R. and W.S.F. are consultants for Merakris Therapeutics, and W.S.F. is a co‐founder and shareholder of Merakris Therapeutics.

## Data Availability

The data that support the findings of this study are available on request from the corresponding author. The data are not publicly available due to privacy or ethical restrictions.
